# Cooling potential for hot climates by utilizing thermal management of compressed air energy storage systems

**DOI:** 10.1038/s41598-022-26666-1

**Published:** 2022-12-21

**Authors:** Abdul Hai Alami, Mehmet Orhan, Rashid Al Rashid, Ahmad Yasin, Ali Radwan, Mohamad Ayoub, Mohammad Ali Abdelkareem, Adnan Alashkar

**Affiliations:** 1 Sustainable and Renewable Energy Engineering Department, University of Sharjah, P.O. Box 27272, Sharjah, United Arab Emirates; 2 Sustainable Energy and Power Systems Research Centre, RISE, University of Sharjah, P.O. Box 27272, Sharjah, United Arab Emirates; 3 Department of Mechanical Engineering, American University of Sharjah, P.O. Box 26666, Sharjah, United Arab Emirates; 4 Department of Mechatronics Engineering, American University of Sharjah, P.O. Box 26666, Sharjah, United Arab Emirates; 5 Materials Science and Engineering PhD Program, American University of Sharjah, P.O. Box 26666, Sharjah, United Arab Emirates

**Keywords:** Energy storage, Renewable energy

## Abstract

This work presents findings on utilizing the expansion stage of compressed air energy storage systems for air conditioning purposes. The proposed setup is an ancillary installation to an existing compressed air energy storage setup and is used to produce chilled water at temperatures as low as 5 °C. An experimental setup for the ancillary system has been built with appropriate telemetric devices to measure the temporal temperature variation, which consequently can be used to calculate the heat transfer and available cooling capacity. The system is compared to commercially available compression cooling air conditioners, and the potential of replacing them is promising, as one ton of conventional cooling can be replaced with a 500-L (0.5 m^3^) air tank at 20 bar operating for an hour. More tanks can be added to extend the operational viability of the system, which is also serving the original purpose of storing energy from grid excess or from solar photovoltaic panels. The thermal management has had the added benefit of increasing the roundtrip efficiency of the storage system from 31.4 to 35.2%, along with handling a portion of the cooling load.

## Introduction

Global population growth rates have witnessed an unprecedented increase in the past decade and is expected to continue until the world reaches a projected 9.7 billion by the year 2050^1^. The direct impact on such an increase will eventually be felt on food, water, and energy supplies. The demand for the latter is estimated to increase by 50% of its value in 2018 by the year 2050. In order to mitigate the energy supply and optimize the energetic, environmental, and economic performance of the power generation systems, it is essential to integrate energy storage systems as they lower the operational cost, reduce the environmental impacts and improve the efficiency of the power generation system. Conventional energy storage systems can be classified into mechanical, thermal, and chemical techniques^2^. While novel energy storage techniques such as thermoelectrical devices^3,4^ and buoyance work^5^ have gained considerable attraction recently.

Compressed air energy storage (CAES) system stores potential energy in the form of pressurized air. The system is simple as it consists of air compressor, reservoir, air turbine, and a generator. At low peak energy demand, energy from a renewable source will power the air compressor and raise the pressure inside the reservoir. At high demand peak, the air leaves the reservoir to the air turbine, which is coupled to a generator, that produces electrical energy and supplies the load. There are three main classifications of CAES systems: Isothermal, Diabatic, and Adiabatic CAES systems. In Isothermal (I-CAES) systems, the operations occur at quasi-equilibrium states that are extremely challenging. In Diabatic (D-CAES) systems, heat generated from the process is wasted to the surrounding environment. Adiabatic (A-CAES) systems, which have the leisure of harboring thermal energy storage (TES) systems are considered to be a median step between I-CAES and D-CAES as they operate with high pressure fluctuations and do not waste the generated heat to the surrounding^6,7^.

Ghalelou et al.^8^ proposed a novel stochastic self-scheduling of renewable energy sources considering thermal units and CAES in the presence of a Demand Response Program (DRP). The results showed that in the case when CAES and DRP are utilized, the total operational cost decreased by 5.1%. Xiaojun et al.^9^ proposed and studied the performance of an integrated CAES/Biomass Integrated Gasification Combined Cycle (BIGCC) system. The integrated system reported a round-trip efficiency of 88.43% and a CAES exergy efficiency of 64.28%. Moreover, Haonan et al.^10^ proposed and investigated a novel isobaric adiabatic compressed humid air energy storage system, that has a single stage multiple synchronous rotating cylinder dual-usage compressor-expander to replace a double multi-stage turbine. The best round-trip efficiency of 66.6% was obtained at a storage pressure of 10 MPa. Chen et al.^11^ proposed a throttling strategy to enhance the inlet pressure of a high-pressure turbine by introducing an ejector. The strategy improved the roundtrip efficiency by 2% and the profit by 21% when compared to conventional A-CAES.

Wen et al.^13^ proposed a technique for recycling the exhaust gas and waste heat of the heat exchange working medium. A four-stage advanced CAES model simulation under steady-state operation circumstances was carried. The results showed that in comparison to no waste heat recovery, the greatest increase in system output power is 4713.72 kW, and the corresponding increase in system efficiency is 7.34%.

Lashgari et al.^14^ investigated the energetic, exergetic, economic, exergoecomonic and environmental performances of a biomass-driven combined heat and power coupled with a CAES system. The numerical results showed that the presence of the CAES unit improved the total efficiency by 67% and the roundtrip efficiency by 12% when compared to the stand-alone system. Moreover, the system demonstrated a levelized cost of electricity of 0.05$/kWh, with a payback period of 2 years and was able to capture as much as 25,764 tonnes/year CO_2_ emission. Sadi et al.^15^ carried a techno-economic analysis of innovative biomass-firing technologies utilized for cost-effective cooling. The simulation results revealed a low cost of cooling 0.031 $/kWh. Arabkoohsar^16^ investigated the performance of a subcooled CAES system integrated with an Organic Rankine Cycle (ORC) utilized for the generation of heat and electricity. The numerical results displayed that presence of the subcooled CAES-ORC system improved the electricity and heat production by 20%. The operation of a tri-generation compressed air energy storage (TCAES) systems has a pre-heating free air expansion in its discharge operation, which means that the expanded air temperature reaches extremely low temperatures (~ −100 °C), that facilitate its usage in district cooling applications. Moreover, the heat generated by the compressors in the charging mode can be utilized in a heating manner through district heating networks. Additionally, according to Rahbari et al.^17^, incorporating an ORC within a TCAES is said to enhance the round-trip efficiency of the system. In their study, they analyzed a 5 MW TCAES-ORC integrated within a wind farm in Denmark, where they found a substantial dependency of the performance on the load itself. In nominal load operation mode, a coefficient of performance (COP) of 1.5 was achieved, which dropped to 1.26 in the off-design operation. Similarly, the exergetic efficiency, the levelized cost of storage and the emissions dropped from 64 to 58%, increased from 141 euro/MWh to 153.7 euro/MWh, and dropped from 4163 to 3640 CO_2_ tons equivalent, respectively. This work experimentally investigates the cooling potential availed by the thermal management of a compressed air energy storage system. The heat generation/rejection caused by gas compression and decompression, respectively, is usually treated as a by-product of CAES systems. The novelty of this work for CAES applications in hot climates, where the electric load demand is mainly due to air conditioning requirements is the mitigation of this demand by correlating the efficient storage parameters with thermal cooling performance of the system. These parameters are experimentally verified, and should be taken into consideration when designing an appropriate heat exchanger to utilize it as a cooling system.

## Methods: system modeling and experimental setup

**Figure 1:**
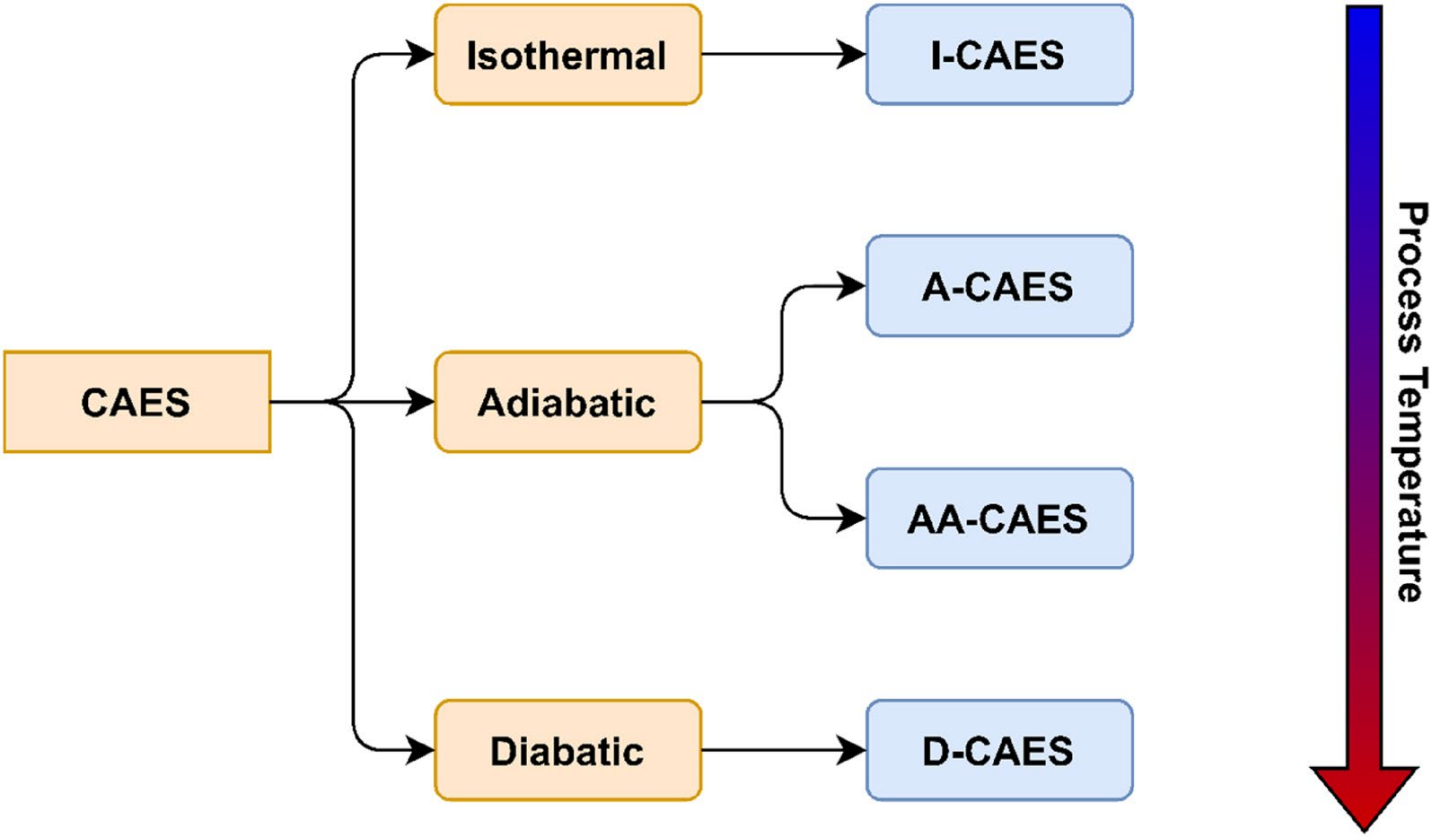
Compressed air energy storage (CAES) systems classification based on thermal management, arranged in terms of process operational temperature.

CAES systems are classified based on operational assumptions related to applied thermal management mechanisms, these types are summarized in Fig. 1. In order to improve the efficiency and reduce the cavitation risk of the equipment during compression and expansion stages, several thermal management techniques can be utilized. When compared to A-CAES and D-CAES, the process temperature in I-CAES is the lowest, since it is based on idealistic assumptions and quasi-equilibrium load application steps. As a result, the temperature of I-CAES can range from 15 to 90 °C, while that of D-CAES and A-CAES spans from 140 to 500 °C and 90 to 700 °C, respectively^18^.

**Figure 2:**
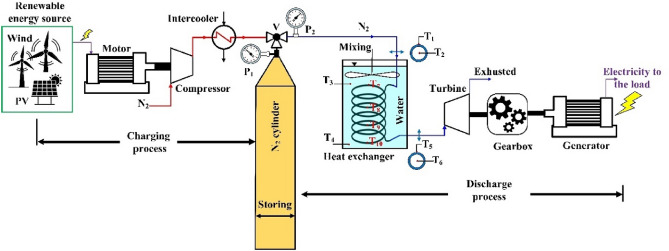
Schematic of the experimental system.

To assess the cooling potential in the expansion process, an experimental setup was constructed containing all the major components of a CAES system. Figure 2 shows the schematic of the experimental setup utilized in the work and the previous work by the authors^21^.

**Figure 3:**
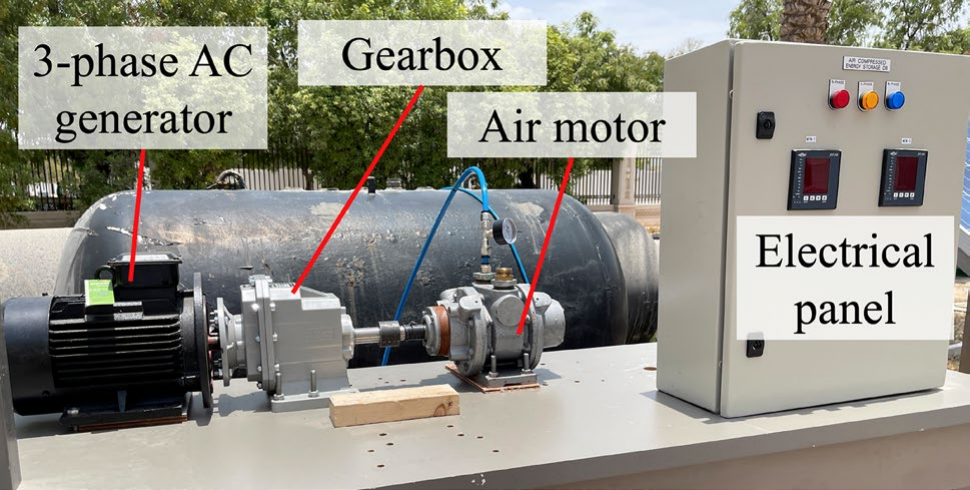
The experimental setup of compressed air energy storage.

The gas (air) sources are commercially available, pre-filled nitrogen tanks of 50 L nominal capacity and nominal pressure of 3000 psi. Since nitrogen makes up 79% of air, the gas properties are taken for nitrogen to simulate the behavior of air. The cylinders are connected to a turboexpander in the form of a 9 hp air-motor, used to convert the potential energy of the compressed air and kinetic energy in the flowing air to mechanical rotational motion. The air motor must operate at an rpm consistent with the constant frequency of the generator’s AC power output. Thus, the air motor is coupled to a 1:8 ratio gearbox, which is in turn is coupled with a 3.5 kW (380 V AC, 5 A) permanent magnet electrical generator. The setup is shown in Fig. 3

To resemble the actual performance and response to loads, the generator powers two 100 W lamps, a drill of 250 W, and a vent fan of 6 W rating and a total supplied current of 1.7A. For the thermal control experiments, a serpentine polyethylene 98 pipe of 9.2 m length and 8 mm inner diameter is used to carry the air into the pneumatic devices. The pipe material has a measured thermal conductivity of 0.3156 W/m K^22^, and around 60% of its length is rolled into four concentric loops of around 40 cm diameter. The loops are submerged in a 23.6 L water bath held in a 53 cm diameter container. The water is continuously stirred to have homogenous temperature distribution. K-type thermocouples are installed at specific locations to measure the fluid's temperature at various locations of the setup. For example, the temperature of the fluid at the pipe inlet and exit are measured at T_1_ and T_2_ locations as shown in Fig. 2. This is done via small holes drilled into the pipes where the thermocouple sensors are inserted. Also, thermocouples are added to the pipe surface to measure the temperature there, and these thermocouples are designated T_3_ and T_4_. Similarly, temperatures of the pipe submerged surface through its length are taken by thermocouples T_5_-T_8_, while water temperature the near pipe's surface and at the center of the water container at locations T_9_ and T_10_ as shown in the Fig. 2. A Graphtec Midi Logger Gl240 was used to obtain the temperature readings of all k-type thermocouples. A second order filter was used to reduce the noise from the sensor readings. The sampling time was set to 1 s, which is equivalent to 1 Hz sampling frequency.

Two experiments have been performed to assess the heat transfer process. First, nitrogen is allowed to exit the tank at a regulated pressure of 140 psi into the air motor with no thermal management, i.e., by not submerging the pipe in water. This helps assess the initial condition of the system and have a baseline of the parameters of the experiment. In the second set of runs, the pipe is submerged in the water bath and the heat transfer from the water is calculated my measuring the temperature change. Air is allowed to expand to a nominal 140 psi exit pressure from the tank and gets lower as the tank pressure drops. The effect of this thermal management on the performance of the storage system is also reported and compared to the case where no thermal management is implemented. During all experimental runs, the nitrogen tanks were mounted on a special fixture and chained to the wall.

For the case at hand, and especially while operating at steady-state, the heat transferred from the water bath to the pipe can be assumed to be constant. It is also well-known that heat exchange along a pipe mainly happens due to mass transfer rather than convection or conduction^23^. Also, the water in the bath is stagnant and thus only conductive heat transfer is expected to occur.

**Figure 4:**
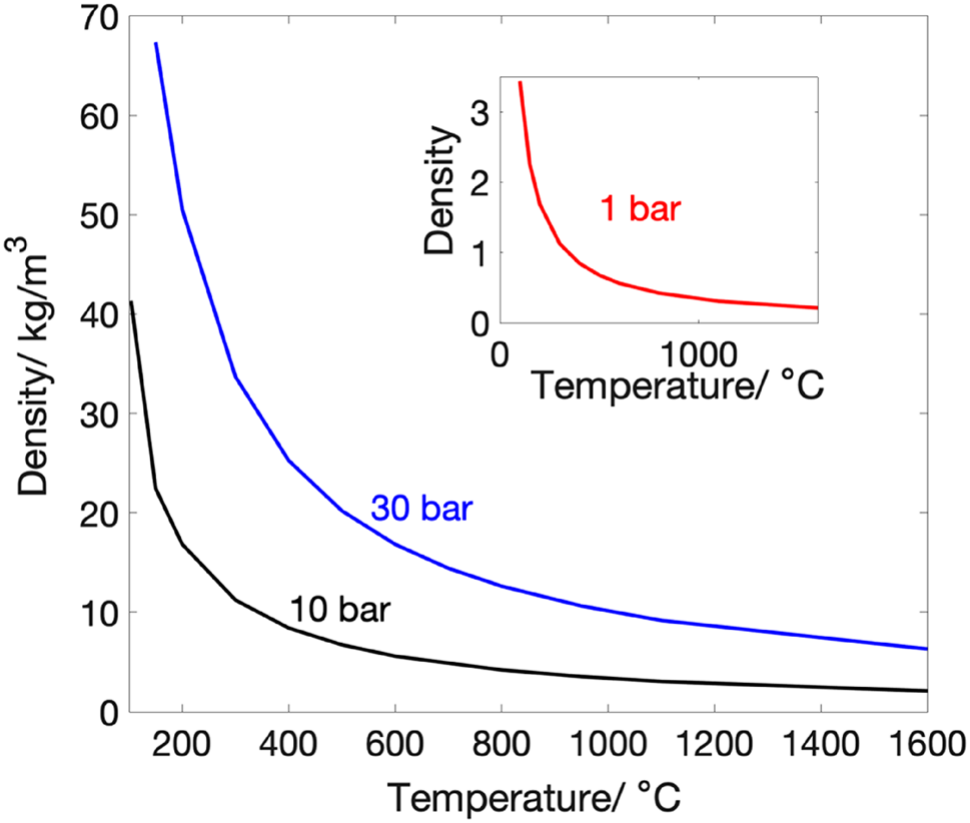
Density of nitrogen with change in temperature and at different pressures.

The potential energy of interactions between molecules in a gas is modest in comparison to their kinetic energy. The region where the ideal gas approximation is applicable is even insignificant at high temperatures and low densities. However, when temperature and density decrease, the proportion of interaction energy to total energy increases, and the characteristics of the gas begin to diverge from those of an ideal gas. Thus, for any CAES system, the operational density of the working fluid is of a great importance as the temperature and pressure are varying during the operation time. Figure 4 shows the variation of density with temperature and at different pressures^24^. It can be seen from the figure that at high pressures the density increases, which is a result of gas molecules packing together and may then approach the behavior of liquid. Hence, because of their thermal energy, the molecules' momentum is lowered, and the particles move closer together until they are close enough for intermolecular interactions to play a substantial influence in defining bulk qualities like viscosity. This may create condensation and air shocks in the piping system and the expander turbine rotor, reducing system efficiency.

## Results and discussion

### System thermal management

**Figure 5:**
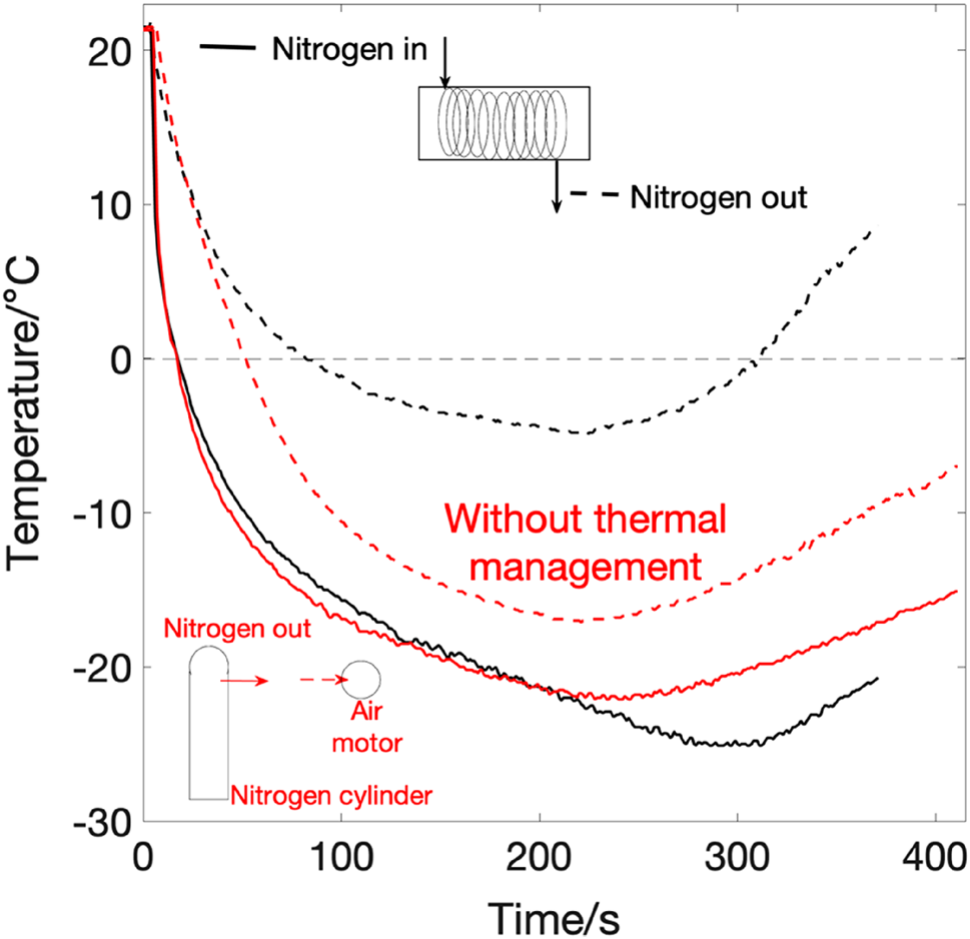
Temperature profile of nitrogen with (black) and witout (red) thermal management in the form of a water bath.

The first run without thermal management was conducted with the nitrogen flow pipe not submerged in water, but exposed to ambient air at room temperature. Results of fluid temperature at inlet and exit are shown in red in Fig. 5. Since heat is being added to the fluid from its surroundings, an increase of 5 °C in the temperature of the fluid is observed between pipe inlet and outlet. The minimum recorded exit temperature at exit points is -17 °C for the fluid and -4 °C for the pipe's surface at inlet and outlet, respectively.

In the second run, 60 percent of the pipe is submerged under water in four loops. The increase of fluid temperature from inlet to exit is measured to be 17.7 °C, where the fluid's inlet, exit temperatures and pipe's surface at exit are − 22.7 °C, − 5 °C and 2.4 °C, respectively. This is also shown in Fig. 5 in black.

**Figure 6:**
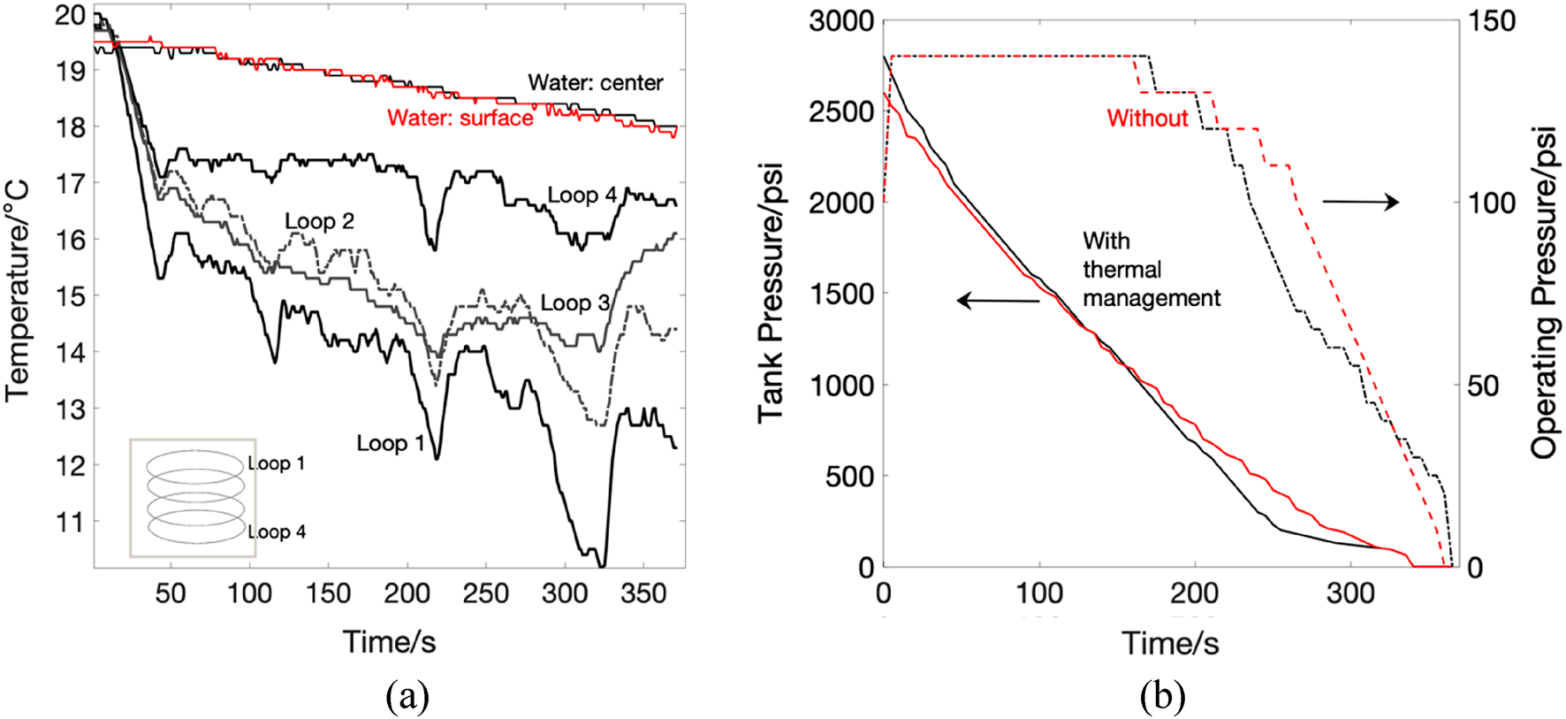
(**a**) Nitrogen pipe surface and water temperature profiles (**b**) pressure values in the storage tank (before regulator) as well as operating pressure (after regulator). Red color is for no thermal regulation and dashed lines are read on operation.

The temperatures of water at the center and near the pipe's surface are shown in Fig. 6a. There is a gradual decrease in the temperature trends as the experiment progresses, with the loop closest to the surface (loop1) showing the lowest temperature, and the loop closest to the bottom (loop 4) showing the highest, since air enters from loop 1. Also, the temperature drop along the submerged pipe's surface is demonstrated. The effect of thermal management can also be seen on the tank's pressure and operating pressures for both runs, as shown in Fig. 6b. The operating pressure was 40% higher when the pressure was not regulated. On the other hand, the performance of the system had appreciable enhancement when thermal management was deployed in the form of a water heat exchanger.

Tables 1 and 2 summarize the recorded operating conditions for both runs, and the maximum amount of heat removed from the system (in kJ), respectively.

**Table 1:** Operating conditions for both runs, with and without thermal management.

System	Operating pressure/psi	Initial water temperature/°C	Final water temperature/°C	Discharge time/s	Pipe area submerged/m^2^	Pipe area exposed/m^2^
Thermal management	140	19.4	17.9	340	0.4139	0.2797
No thermal management	140	–	–	385	–	0.6936

**Table 2:** Maximum heat removed with heat exchanger deployment and minimal temperatures.

System	Minimum fluid inlet temperature/°C	Minimum fluid exit temperature/°C	Q/kJ
With Heat exchanger	− 25	− 5	148.04

### CAES system performance

**Figure 7:**
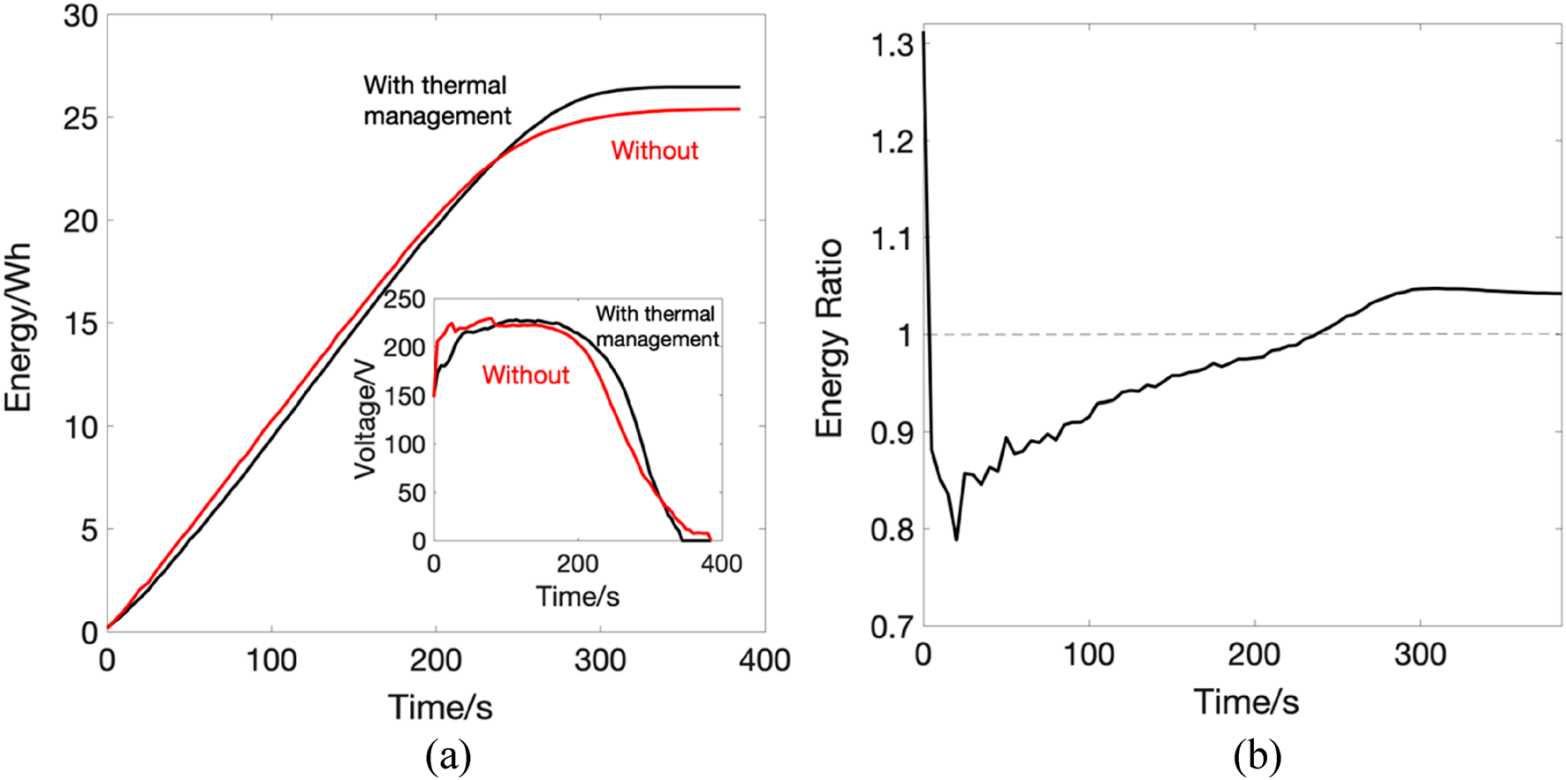
(**a**) Energy plots for the CAES system with (black) thermal management and without (red), inset shows the overall enhancement of the voltage (**b**) the energy enhancement ratio over the experimental run time.

**Figure 8:**
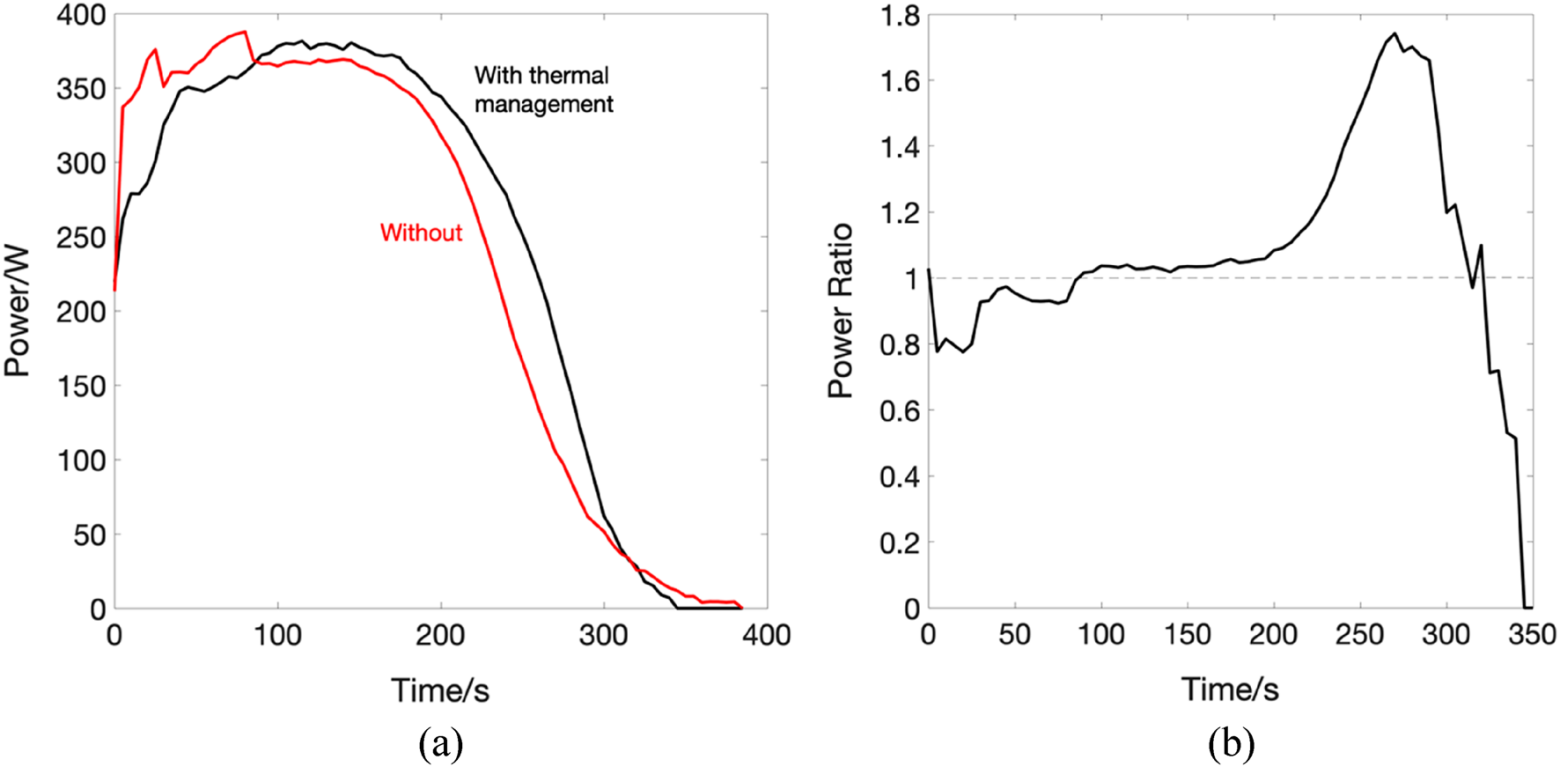
(**a**) Power plots for the CAES system with (black) thermal management and without (red) (**b**) the power enhancement ratio over the experimental run time.

The effect of thermal management on the storage system can be identified by the enhanced measured parameters such as the generator output voltage, energy and power. In general, the addition of the heat exchanger has enhanced the system's energy and power, as can be seen from Fig. 7 and Fig. 8, respectively. The enhancement factor is calculated as a ratio between values measured with thermal management divided by their counterparts measured without thermal management.

From the results shown, it noticeable that the system performance has benefited from adding the heat exchanger. The total energy produced by the system for the given loads is 26.4 Wh in the case of the heat exchanger, and 25.39 Wh without. Furthermore, looking at the power graph it can be noticed that the performance of the system is steadier and a smoother transient at the beginning of the experiment, with minimum spikes for the heat exchanger experiment, is exhibited, compared to its counterpart. The operating voltage was maintained to the nominal operational value of 220 V at 140 Psi when increasing the temperature of the fluid entering the air motor, compared to the case without heat exchanger where the voltage falls below the nominal value, see the inset of Fig. 7a. The roundtrip efficiency of any storage system determines its overall performance. The fraction of electrical work that is saved and recovered later is termed as roundtrip efficiency. With a higher roundtrip efficiency, less energy is wasted throughout the storage process. The roundtrip efficiency is given as:

1 $${\eta }_{r}=\frac{{E}_{produced}}{P\cdot {V}^{1.4}}$$

where ${E}_{produced}$ is the electrical energy produced by the system in joules, *P* is the working pressure and *V* is the storage tank volume.

The round-trip efficiency of the system increased by 3.8% with the heat exchanger added. More details are shown in Table 3.

**Table 3:** Summary of CAES system performance with and without thermal management.

System	${\eta }_{roundtrip}/\mathrm{\%}$	Energy/Wh
With thermal management	35.2	26.46
Without thermal management	31.4	25.39

### Experimental Error Analysis

The sources of possible error in the experimental measurements are traced to the equipment. The temperature thermocouples and logger as well as the electrical power measurement devices are the equipment used the most. According to the product specifications of the Graphtec Midi Logger Gl240, the accuracy and tolerance of the measured temperature for a K-type thermo-couple is given by the following relation:

2 $${\text{Measurement\,accuracy}} = \pm (0.05\% \,{\text{of\,displayed\,value}} + 1.0^\circ {\text{C}})$$

**Figure 9:**
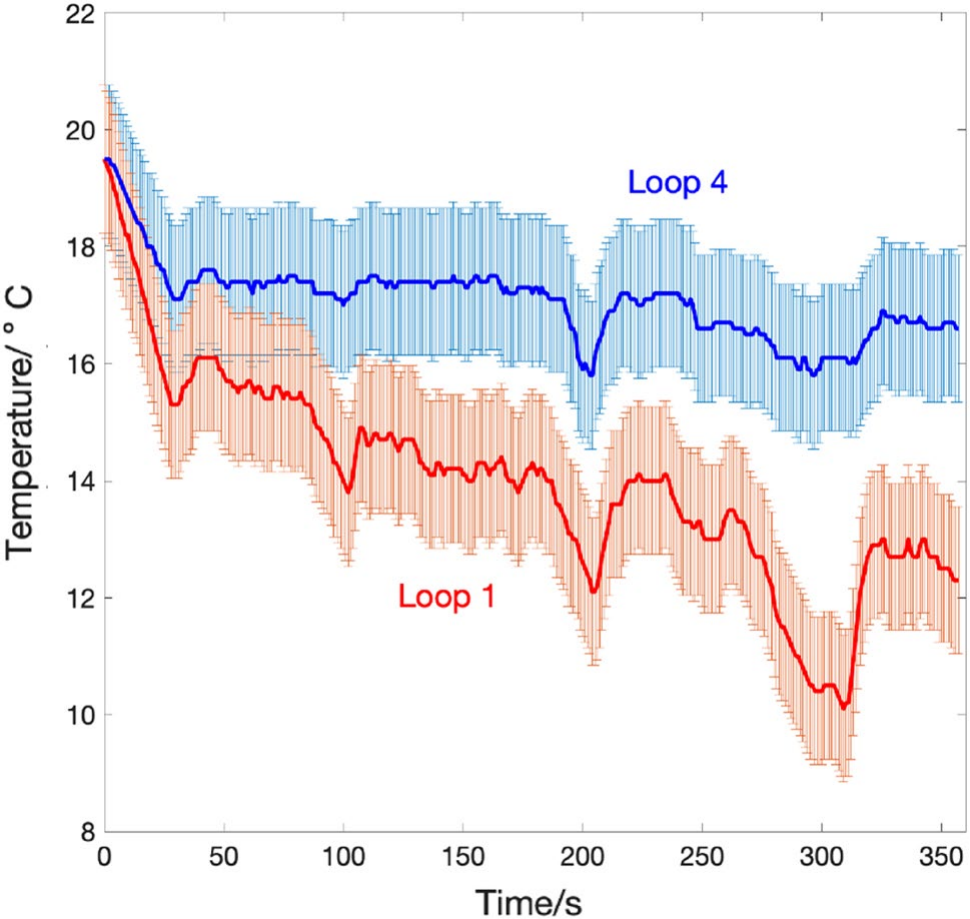
Error analysis of temperature variation during system discharge for Loop 1 versus Loop 4.

Figure 9 shows the variation of temperature measured for four consecutive runs and the error bars were drawn as the standard deviation of the central line, which is the average power value, and by taking into account the Gl240 logger accuracy (Eq. 2). The results show good repeatability since the data logger has high resolution, and the error bars show the significant temperature difference after 150 s of operation, which indicates the decoupling of the two temperature readings of the loops.

**Figure 10:**
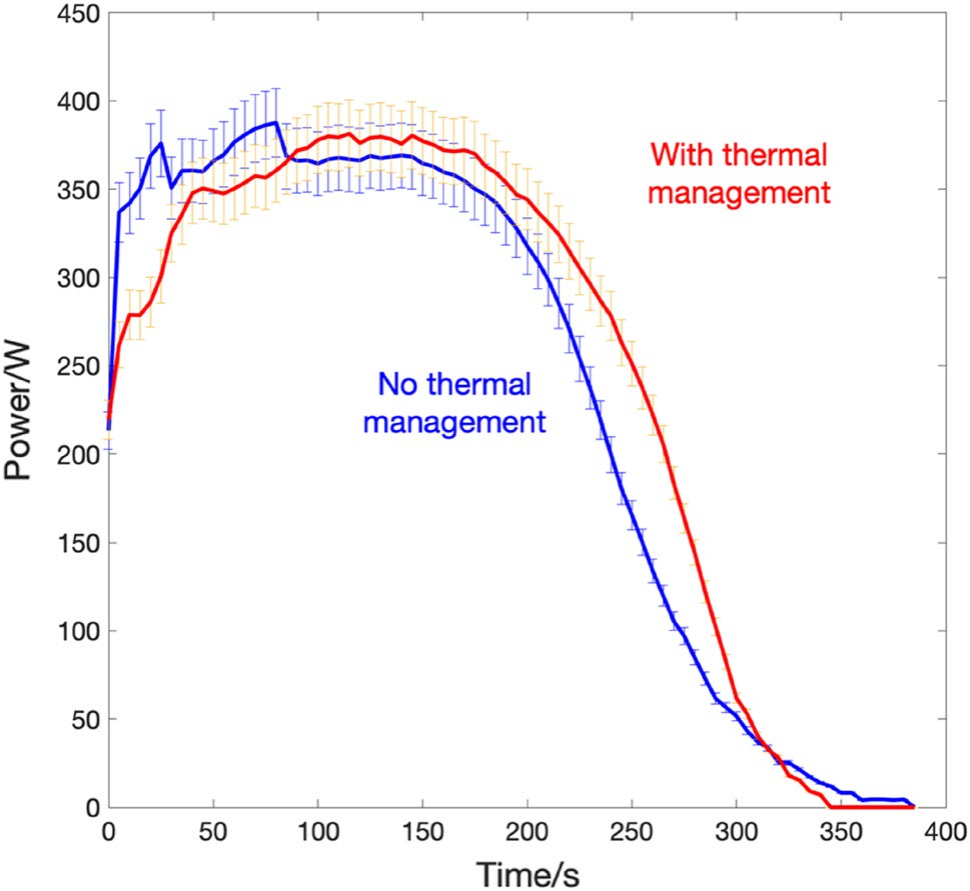
Error analysis of power run of the compressed air system with (red) and without (blue) thermal management.

A similar approach was used for the power generated analysis. The variation in the generated power for four consecutive and the pertinent error bars are shown in Fig. 10. Since the steady state is reached around the 200 s mark, the results show a significant enhancement once the thermal management is in place. It is worth noting that the system runs shown utilize a single nitrogen tank, and for continuous operation, tanks will have to be connected in parallel to ensure uninterrupted supply^21^.

### Comparison with commercial compression cooling setup

**Figure 11:**
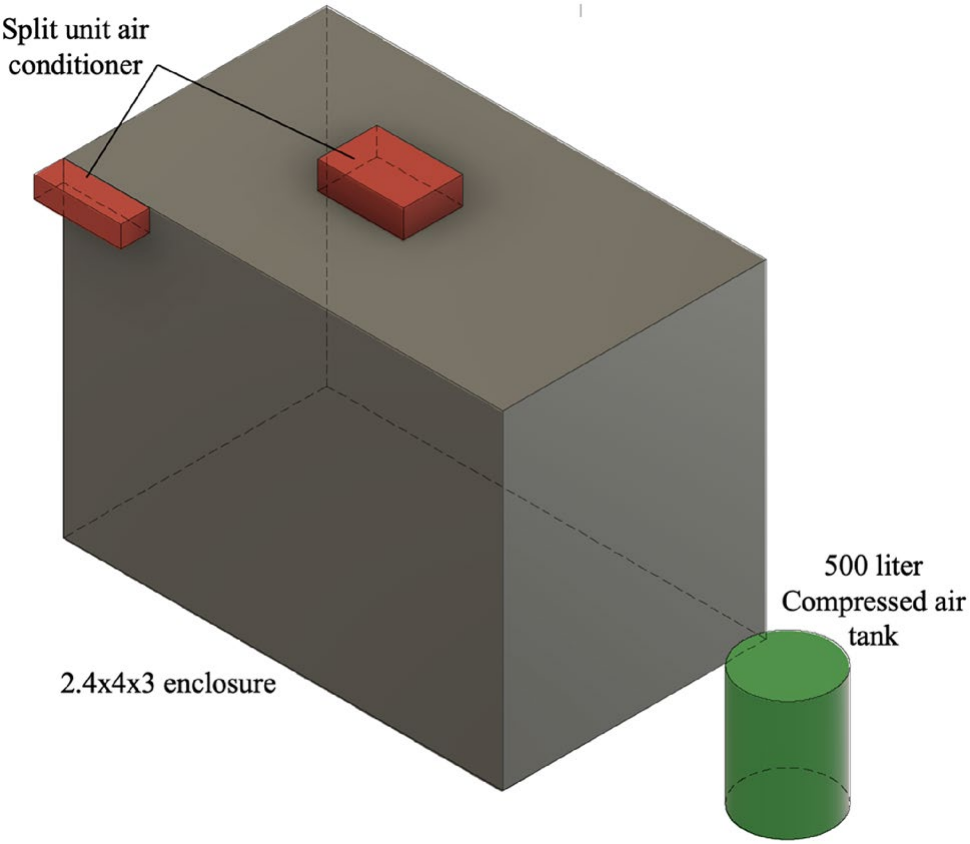
To-scale representation of a typical 1-ton air conditioning split-unit (red box objects) compared to the air storage tank required for equivalent expansion cooling volume of 500 L storage at 20 bar storage pressure.

The cooling load calculation was facilitated using an HVAC online calculator from ServiceTitan^25^. The calculations were made for a small room, shown in gray in Fig. 11, with a size of 3 × 4 × 2.4 m with good insulation, medium solar incidence and typical door and windows fit and seal, resulting in a calculated cooling load of 3600 BTU or 0.3 cooling tons. Typically, at this rate the minimum available in market capacity is 12,000 BTU/1 ton/3.52 kW.

Considering the total operating time of one 50-L cylinder is around 380 s till complete discharge. It is noted that the first 80 s of operation are dedicated to bringing the air motor up to the operating speed. It is observed that the tank pressure becomes insufficient to provide the required operating speed after around 200 s from the dead stop of the motor. This leaves the useful tank discharge time to be around 100 s at steady-state conditions. For this useful time, the rudimentary heat exchanger absorbs 148 kJ/0.0411 kWh/140 BTU/0.01166 ton, meaning that ten 50-L tanks are required, or a total volume of 0.5 m^3^. This is shown as the green cylinder in Fig. 11. Excluding the start and end of the run, the power will be supplied by the generator at nearly 375 W with thermal management and 345 W without thermal management for 3320 s. That is equivalent to 345.8 Wh and 318.16 Wh respectively (3320/3600 × 375&345).

## Conclusion

This work examined the potential of using the thermal management of compressed air energy storage systems to provide an alternative to conventional cooling methods. During air compression in tanks (system charge) and air expansion (system discharge), heat must be removed and added to air, respectively, to ensure efficient operation from a thermodynamical point of view, as well as, device protection from two phase flow (water vapor condensation and causing cavitation). The opportunity to use the expansion process to chill water for air conditioning purposes is explained, and the amount of heat is calculated during runs for the energy storage system. The expansion system operation was compared with that of a 12,000 Btu compression cooling cycle to remove the cooling load from an enclosure of 2.4 × 4 × 3 m^3^ dimensions. The physical footprint of the compressed air system is dominated mainly by the air storage tanks, and in this case 500 L tank storage is required to operate a 9 hp air motor that is coupled to a 380 V generator. This ensures identical operation for an hour, which means that more tanks could be used or air pressure should be replenished within the cylinder for continuous operation. The advantages of the proposed system are numerous, chief among which is that the cooling is side product of another process for energy storage where thermal management can be used for the advantage of a user that is mainly in a hot climate. This reduces the reliance on conventional air conditioning units, which are the major consumers of electrical power. Also, the energy storage process has seen around 4% enhancement in roundtrip efficiency by employing the air heating by chilling the water for air conditioning purposes. The proposed system is cheap and requires no special refrigerants or power intense compressors.

## Data Availability

The datasets used and/or analyzed during the current study are available from the corresponding author on reasonable request.
